# Supplementary material for: Exchanging Ohmic Losses in Metamaterial Absorbers with Useful Optical Absorption for Photovoltaics
Source: Sci Rep. 2014 May 9;4:4901. doi: 10.1038/srep04901 (PMC4014987; doi:10.1038/srep04901)
Supplement: Supplementary Information — Exchanging Ohmic Losses in Metamaterial Absorbers with Useful Optical Absorption for Photovoltaics [file srep04901-s1.pdf]

Supplementary Information: Exchanging Ohmic Losses in Metamaterial Absorbers with Useful Optical Absorption for Photovoltaics

Ankit Vora[1], Jephias Gwamuri[2], Nezih Pala[3], Anand Kulkarni[1], Joshua M. Pearce[1,2], and Durdu Ö. Güney[1,*]

[1]Department of Electrical and Computer Engineering, Michigan Technological University, Houghton, MI 49931, USA
[2]Department of Materials Science and Engineering, Michigan Technological University, Houghton, MI 49931, USA
[3]Department of Electrical and Computer Engineering, Florida International University, Miami, FL 33174 USA
[*] Corresponding Author: dguney@mtu.edu

Previous applications[1, 2] of the BSDT.

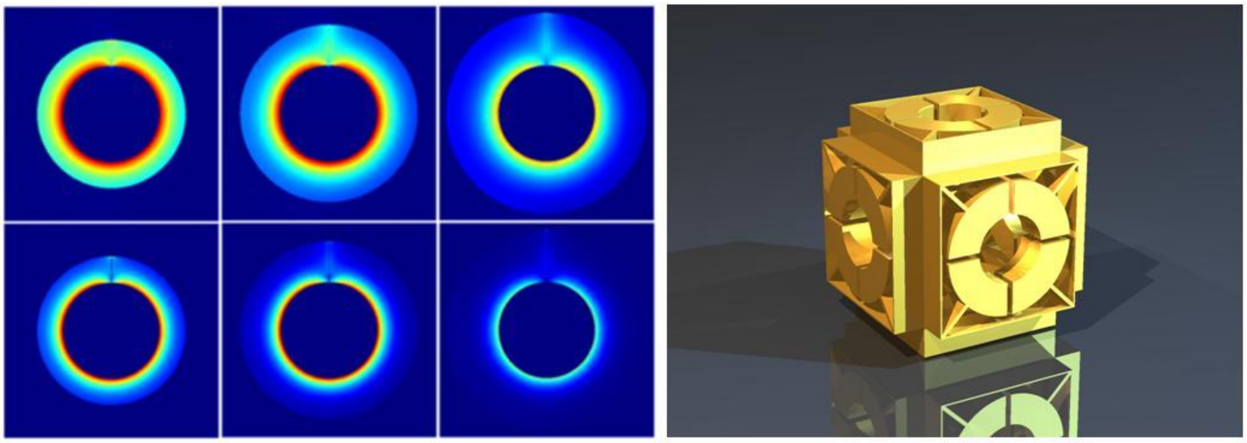

(a) (b)

Figure S1 (**a**) BSDT can be used to significantly reduce the geometric skin depth by increasing the radius of curvature of the surfaces where the resonant current flows. Thus the resonant current cannot penetrate deep into the metal; rather it is tightly bound to the surface. The top (bottom) panel from left to right shows the current (power loss) density distribution for split-ring-resonators with increasing radius of curvature which is achieved by making the rings thicker and bulkier. Only two-dimensional cross-sections are shown. The current density ranges between 0 (dark blue) and 6×10^{14} A/m^2 (dark red) and the power loss density ranges between 0 (dark blue) and 4.5×10^{21} W/m^3 (dark red). [modified from Figure 6 in "Guney, D. O., Koschny, T. & Soukoulis, C. M. Reducing ohmic losses in metamaterials by geometric tailoring. *Phys. Rev. B* **80**, 125129 (2009)"] (**b**) This effect can be further employed to design a fully connected three-dimensionally isotropic negative index metamaterial at optical frequencies by assembling the split-ring-resonators in the form of a cubic unit cell. The electrical interconnects do not short circuit the split-ring resonators due to the tight confinement of the loop currents. [Figure 2(b) in D. O. Guney, Th. Koschny, and C. M. Soukoulis, Intra-connected three-dimensionally isotropic bulk negative index photonic metamaterial, *Opt. Express* **18**, 12348 (2010).]

Metamaterial absorbers with $In_xGa_{1-x}N$ spacers.

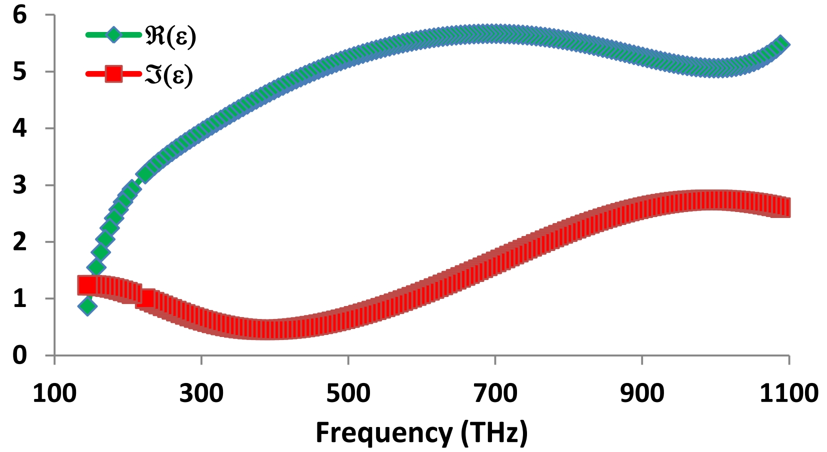

Figure S2 Experimental complex permittivity data for $In_{0.54}Ga_{0.46}N$.

Exchanging Ohmic losses at the infrared spectrum.

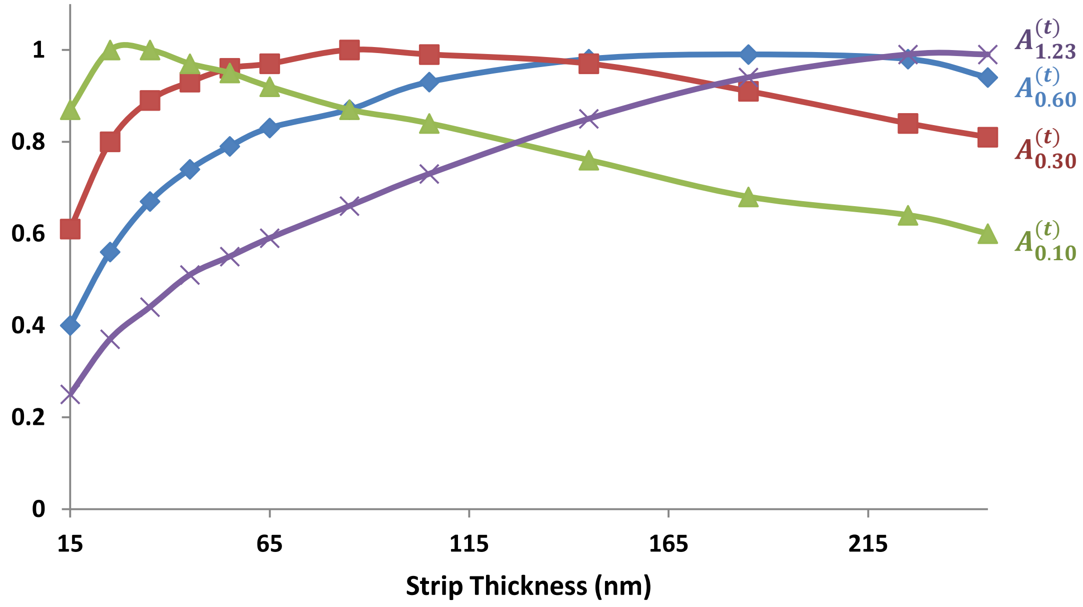

Figure S3 Total absorbance (i.e., sum of the absorbance in the spacer and metallic regions) $A_j^{(t)}$ for different $\Im(\varepsilon)$ values j of the spacer versus t_s corresponding to Fig. 4(a).

Scaling the Ohmic loss exchange toward the red edge of the visible spectrum.

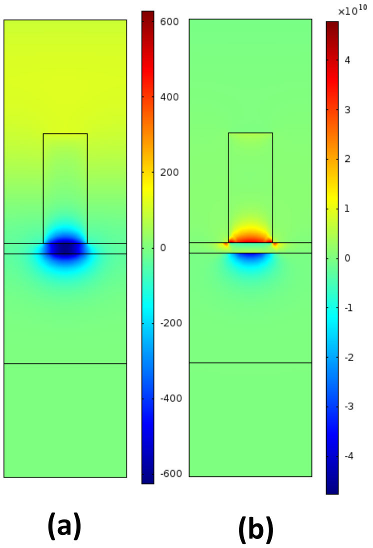

Figure S4 (**a**) H_z (A/m), and **(b**) J_x (A/m^2) for the absorber in Fig. 5(d) at the peak total absorbance.

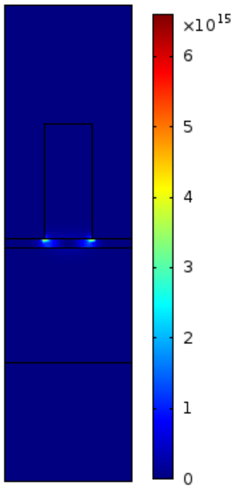

Figure S5 Power loss density (W/m^3) distribution for the near-perfect absorber in Fig. 5(g) for the second selected frequency of 387THz in the $In_{0.54}Ga_{0.46}N$ data (i.e., Point 2 in Table 1). Significant portion of the power (i.e., over 70%) is absorbed inside the $In_{0.54}Ga_{0.46}N$ layer.

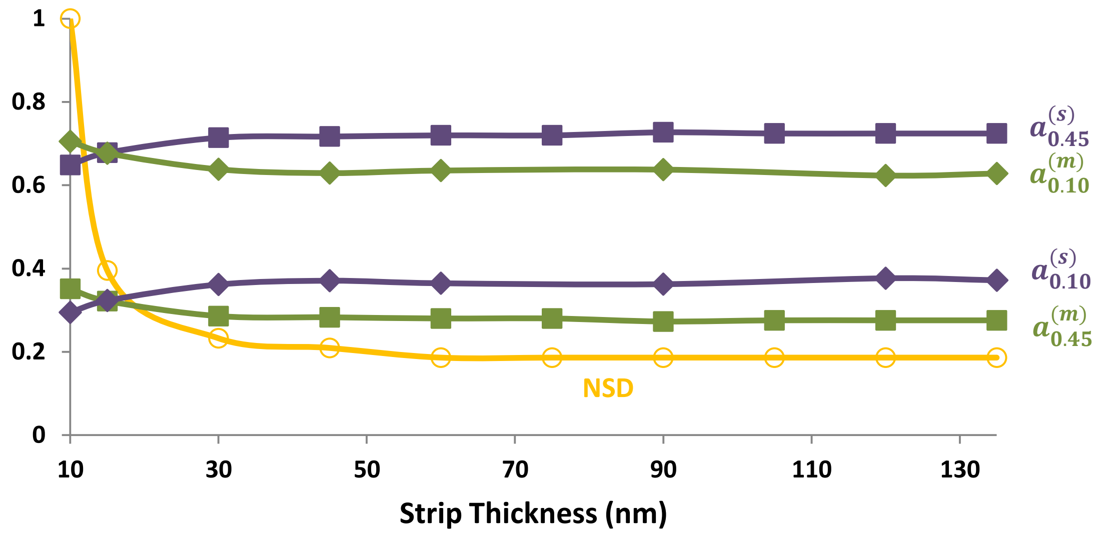

Figure S6 The percentage of total absorbance $a_j^{(i)}$ in different regions of the metamaterial absorber corresponding to Fig. 6(a) and NSD versus t_s.

Ohmic loss exchange at the ultraviolet frequencies. The resonant peaks in the neighborhood of Point 4, 1000THz, are displayed in Fig. S7. We observe four resonant peaks, one of which has near-perfect absorption at 950THz. Magnetic field distributions for these resonance peaks from left to right are shown in Figs. S8(a)-(d), respectively. We will call these modes as Modes I-IV. All the resonant modes except the near-perfect absorber mode (i.e., Mode II) have significant reflectance. The Modes I and IV have additionally significant transmittance, which can be minimized by increasing the thickness of the ground plate. Thus, the part of the transmitted power can be converted to absorbed power.

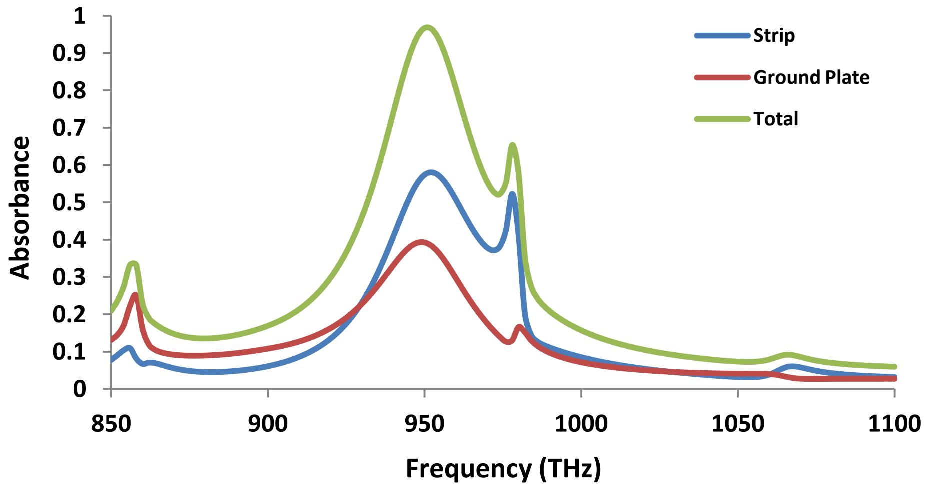

Figure S7 Absorbance spectra in the neighborhood of Point 4 for different parts of the absorber. The second resonance at 950THz gives near-perfect absorbance. All the geometric parameters are listed in Table 1.

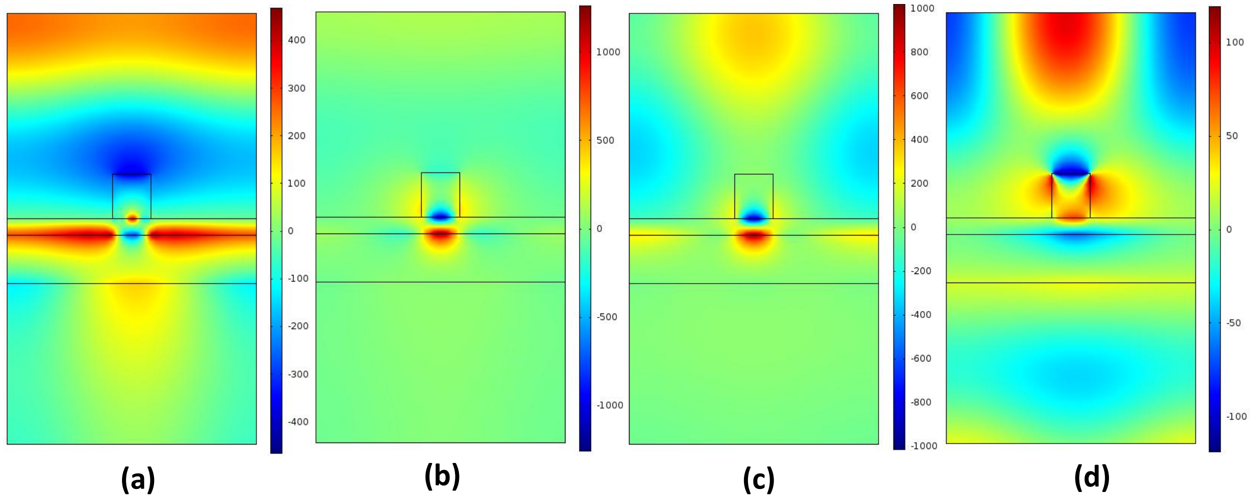

Figure S8 H_z (A/m) for (**a**) Mode I, (**b**) Mode II, (**c**) Mode III, and (**d**) Mode IV, corresponding to the four resonant peaks, respectively, in Fig. S7.

Unlike previous Points 1-3, we notice all the modes in Figs. S8(a)-(d) have field confinement at both metal-spacer and metal-air interfaces. However, to use BSDT more effectively for exchanging the metallic losses with optical absorption in the semiconductor layers, surface plasmons are desired to reside at only metal-semiconductor interfaces, so that they can be pushed further toward the semiconductor surface. Fortunately, here, large $\Im(\varepsilon_4)$ help achieve significant absorbance in the semiconductor layer despite surface plasmons residing at the metal-air interfaces. In general, interfacing metals with other lossy materials, especially if surface plasmons reside at those interfaces, should be avoided to maximize the absorption localization change from metals to semiconductor layers.

Figs. S7 and S9 show how BSDT, as applied to metal-spacer interface only, affects the metallic losses for each resonant mode. For Mode I [see Fig. S8(a)], increasing the metallic thicknesses gives a small decrease in the metallic loss from near 35% to below 25%. Even if we do not start from a perfect absorber (i.e., there exists transmittance and reflectance) and there are surface plasmons residing at metal-air interfaces, BSDT still gives reduction in metallic losses. Because the surface plasmons mainly reside at the interfaces [i.e., interfaces parallel to the metal-spacer interfaces, which asymptotically approach to the surface of a bulk (or semi-infinite) metal under the way we apply the BSDT], which comply with the BSDT. The near-perfect absorber mode, Mode II [see Fig. S8(b)], supports also weak surface plasmon oscillations at the vertical metal-air interfaces which are not accounted by BSDT. Therefore, this limits the reduction in the metal regions to about 70% absorbance (i.e., about 26% decrease in metallic losses). In comparison with the perfect (or near-perfect) absorbers in Points 1-3, this mode gives the least reduction in the metallic absorbance using BSDT.

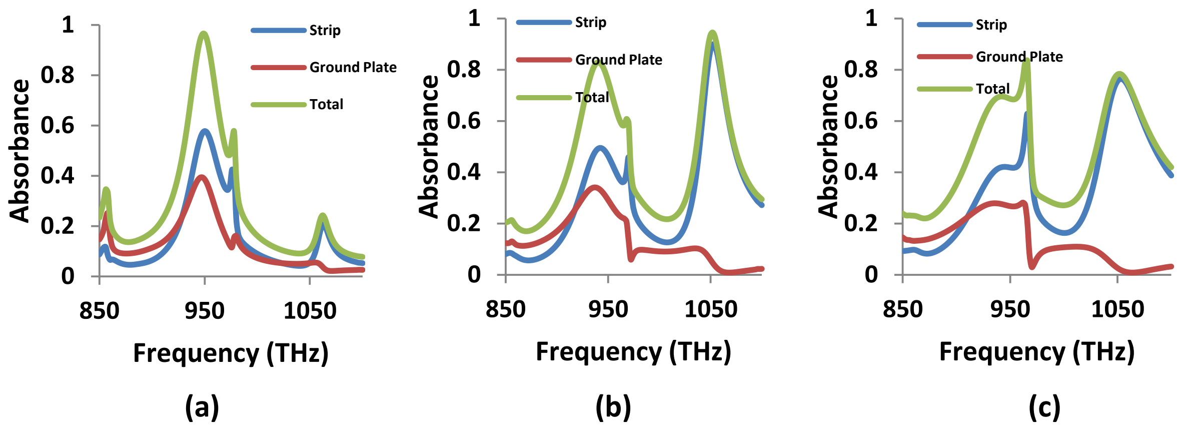

Figure S9 Absorbance spectra in the neighborhood of Point 4 when (**a**) $t_s = t_g = 60$nm, (**b**) $t_s = t_g = 90$nm, and (**c**) $t_s = t_g = 105$nm. All the other parameters are kept fixed and the same as in Table 1.

The absorbance trend for Mode III [see Fig. S8(c)] is quite different than previous modes. The metallic absorbance first decreases from above 65% at 55nm strip thickness to about 55% at 60nm thickness. Then, the absorbance reaches above 80% when the metal thicknesses become 105nm. In contrast with all the other previous modes, here, the absorbance trend is not monotonic. Because when the metal thicknesses approach 105nm, a new resonance starts to emerge at about the same frequency as Mode III. This new mode can be distinguished from Mode III by carefully comparing Fig. S8(c) with Fig. S8(b) and Fig. S10. Notice that Fig. S8(c) has the reminiscence of Mode II in Fig. S8(b) due to the close proximity. Moreover, the magnetic field distribution at the horizontal strip-air interface in Fig. S10 manifests a strong surface wave which does not exist in Fig. S8(c). To distinguish this new mode from other modes, we will call Mode V. The non-monotonic trend that we see here shows that BSDT does not always result in reduced losses, especially when it leads to a resonance condition. Such a non-monotonic trend is also observed for Mode IV [see Figs. S7, S8(d) and S9]. The metallic losses increase until near-perfect absorbance is reached at 90nm metal thicknesses. Above this thickness, BSDT gives reduction in the metallic losses from about 95% to below 80%. Similar to Mode II, the existence of surface waves at the vertical metal-air interfaces limit the effectiveness of BSDT for Mode IV.

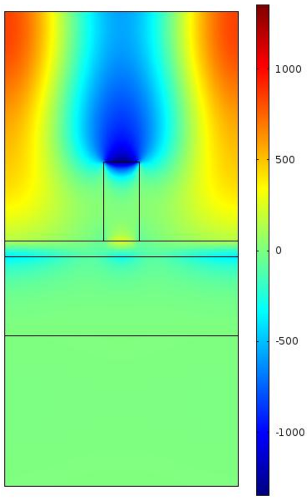

Figure S10 H_z (A/m) for Mode V at 966THz (i.e., absorbance peak for the strip) in Fig. S9(c).

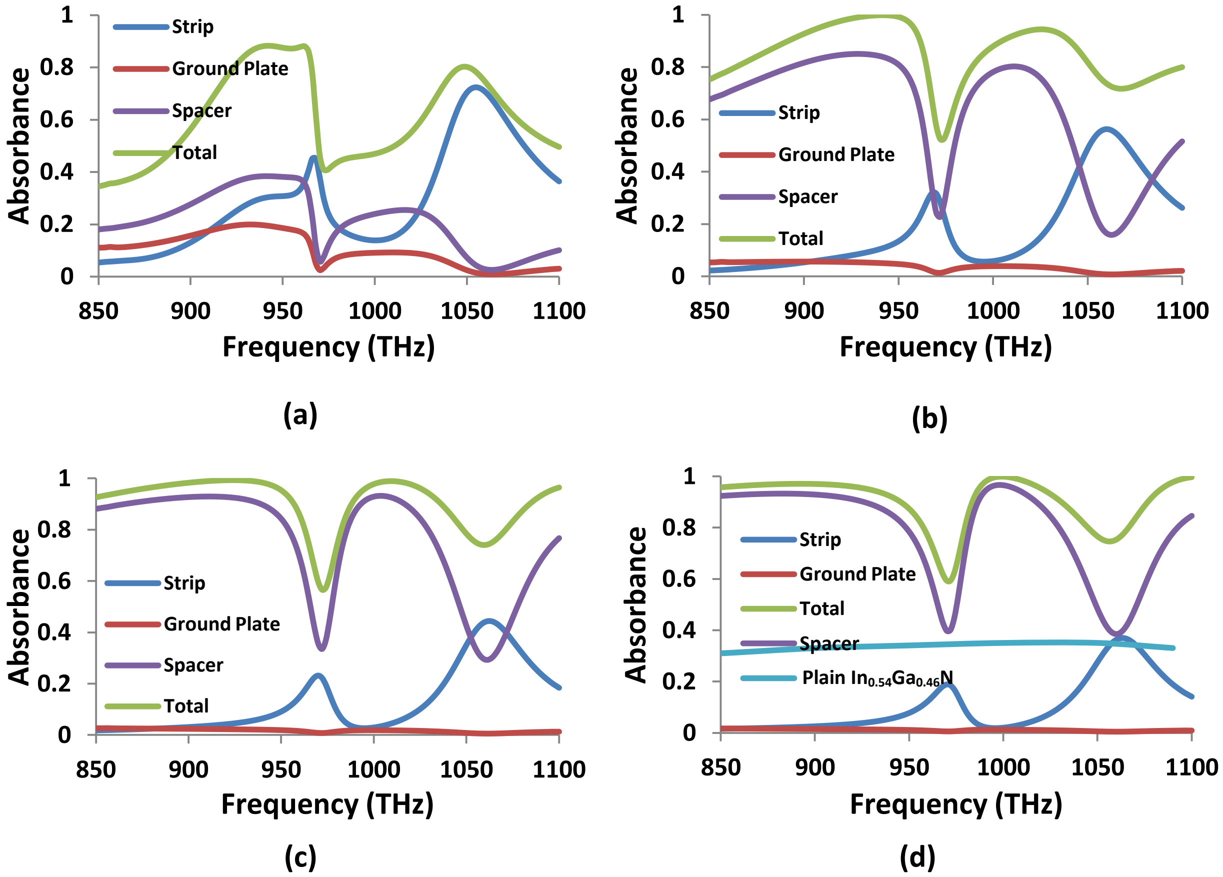

Figure S11 Absorbance in different parts of the absorber achieved by the BSDT process described in Fig. S9 followed by the gradual incorporation of the true complex permittivity such that (**a**) $\Im(\varepsilon) = 0.1$, (**b**) $\Im(\varepsilon) = 0.74$, (**c**) $\Im(\varepsilon) = 1.74$, and (**d**) $\Im(\varepsilon) = \Im(\varepsilon_4) = 2.74$. The total absorbance in plain $In_{0.54}Ga_{0.46}N$ layer of the same thickness (i.e., $d = 20$nm) is also shown for comparison based on the experimental complex permittivity data in Fig. S2.

Having applied the BSDT, Fig. S11 shows how the restoring $\Im(\varepsilon_4) = 2.74$ affects the absorbance in the spacer. Fig. S12 shows the magnetic field distribution for the three absorbance peaks in Fig. S11(a) corresponding to the strips. Comparing these field distributions with Figs. S8(b), S10, and S8(d), respectively, and investigating Fig. S11, we observe that Modes IV and V are the only modes which survive the imaginary part of the permittivity of the semiconductor. Although Mode II persists at low imaginary part, it cannot survive the true value of the $\Im(\varepsilon_4)$. This is because, if we carefully investigate the field distribution for the Modes IV and V, they have significant portion of the field enhancement at metal-air interfaces compared to Mode II, where most of the field enhancement is at the metal-spacer interface. Similarly, Mode I should also have most of the field enhancement at the metal-semiconductor interface as can be seen from Fig. S8(a). Therefore, restoring the imaginary part of the semiconductor permittivity to its true value increases absorbance significantly in the neighborhood of Point 4 except in the frequency regions which mainly correspond to Modes IV and V. The absorbance in the semiconductor layer at Point 4 exceeds 95%. The absorbance in the semiconductor layer at the frequency position, where originally the near-perfect absorber mode (i.e., Mode II) existed, reaches about 80%. In contrast, the total absorbance in plain $In_{0.54}Ga_{0.46}N$ layer for the same thickness (i.e., $d = 20$nm) is between the ranges of $31\% - 35\%$ for the available experimental complex permittivity data in Fig. S2. Based on these important observations, we should also note that embedding metallic parts of the perfect absorbers in the semiconductors may help increase absorbance in the semiconductor by eliminating undesired surface waves at metal-air interfaces especially when higher order modes are of concern.

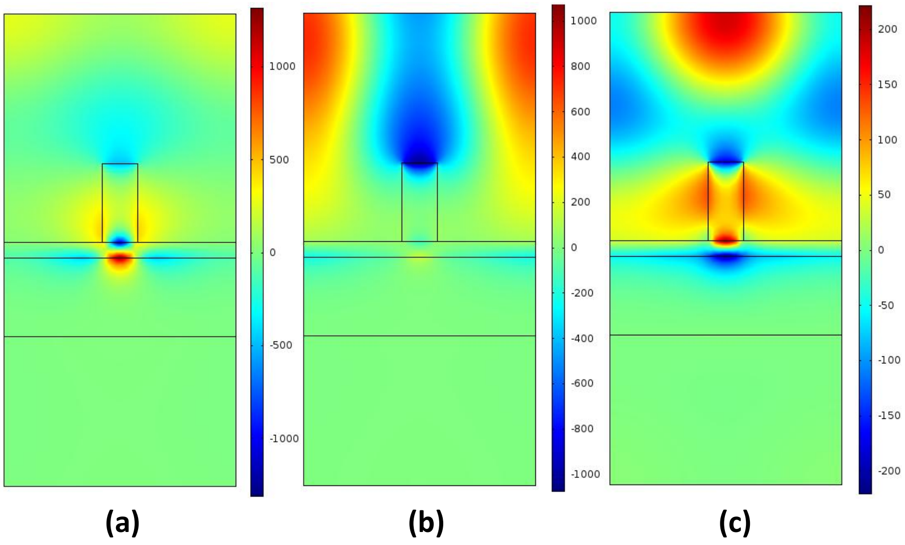

Figure S12 H_z (A/m) profile at the absorbance peaks of the strip in Fig. S10(a) for (**a**) Mode II, (**b**) Mode V, and (**c**) Mode IV.

Due to the small frequency difference between Points 4 and 5, all the resonant modes above, including near-perfect absorber mode (i.e., Mode II), can be easily blue-shifted toward Point 5 by adjusting geometric parameters. When we adjust the geometric parameters as listed in Table 1, the near-perfect semiconductor absorbance peak at Point 4 [see Fig. S11(d)] blue-shifts to Point 5 with about the same absorbance level of 95% in the semiconductor layer. The total absorbance in plain $In_{0.54}Ga_{0.46}N$ layer is between the ranges of $33\% - 35\%$ for the available experimental complex permittivity data in Fig. S2. Fig. S13 shows the effect of BSDT alone on the resonant modes, while Fig. S14 shows the effect of the lossy spacer (i.e., semiconductor). As expected, under both effects the overall behavior of the resonant

modes is clearly similar to the case for Point 4 (i.e., compare Fig. S13 with Figs. S7 and S9; and Fig. S14 with Fig. S11).

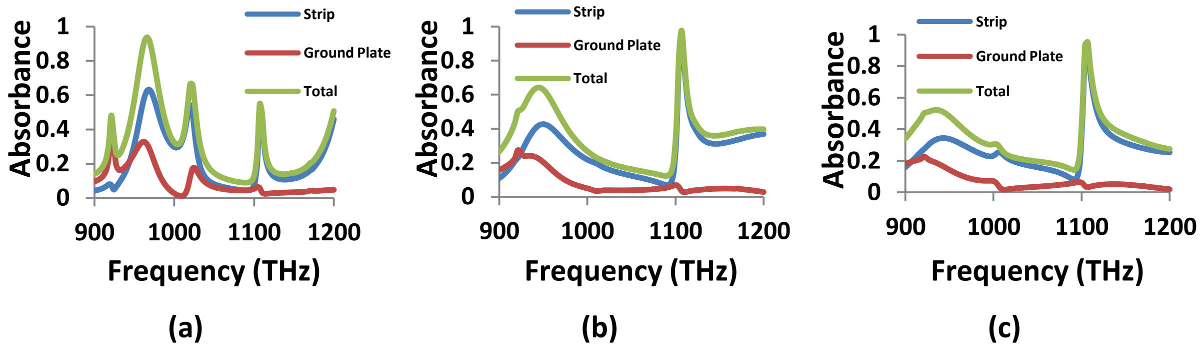

Figure S13 (**a**) Absorbance spectra in the neighborhood of Point 5 for different parts of the absorber. The second resonance gives near-perfect absorbance. All the geometric parameters are listed in Table 1. (**b**) Absorbance spectra when (**a**) $t_s = t_g = 70$nm, (**b**) $t_s = t_g = 95$nm, and (**c**) $t_s = t_g = 105$nm. All the other parameters are kept fixed and the same as in Table 1.

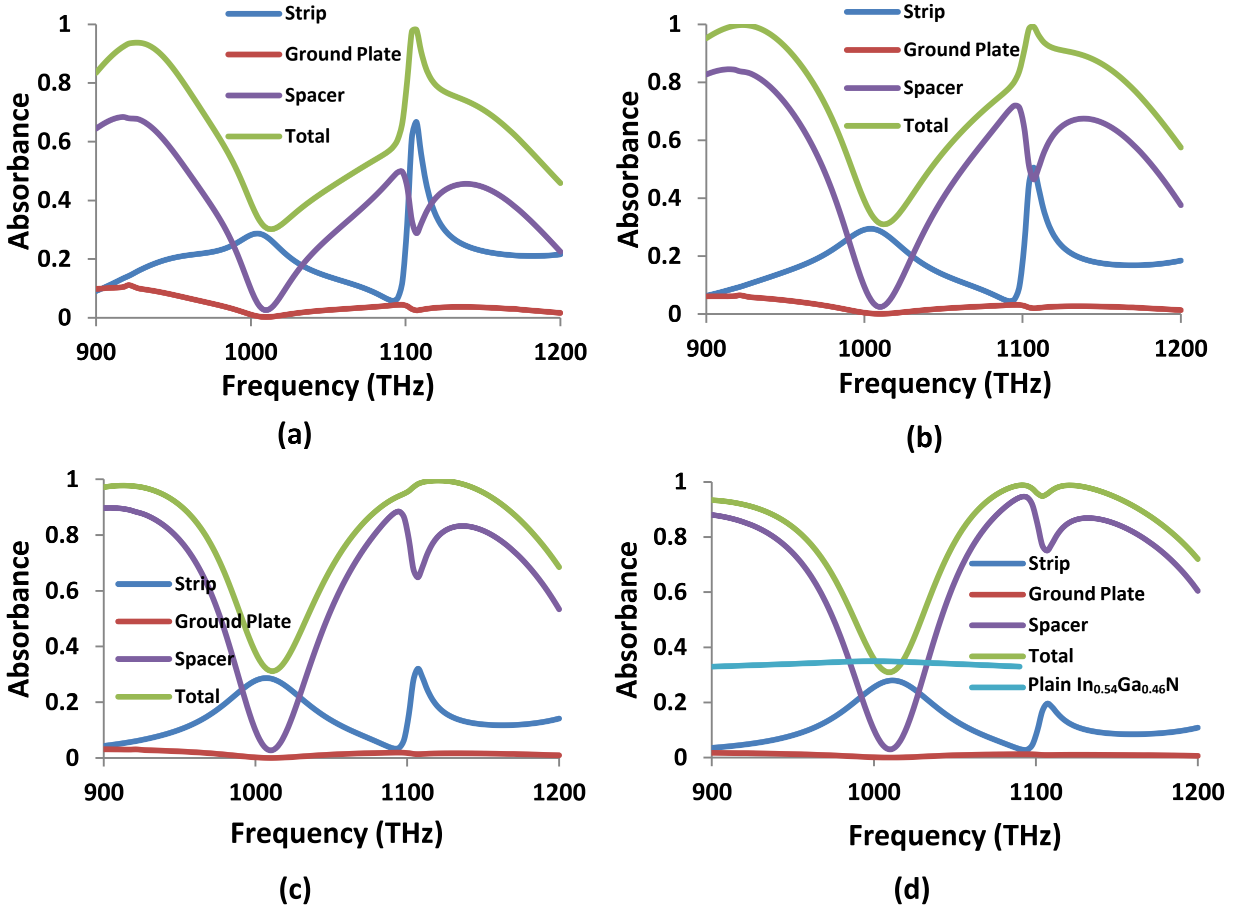

Figure S14 Absorbance in different parts of the absorber with the parameters same as in Fig. S13(c) except for (**a**) $\Im(\varepsilon) = 0.4$, (**b**) $\Im(\varepsilon) = 0.8$, (**c**) $\Im(\varepsilon) = 1.6$, and (**d**) $\Im(\varepsilon) = \Im(\varepsilon_5) = 2.6$. The total absorbance in plain $In_{0.54}Ga_{0.46}N$ layer of the same thickness (i.e., $d = 18$nm) is also shown for comparison based on available experimental complex permittivity data in Fig. S2.

References

1. Guney, D. O., Koschny, T. & Soukoulis, C. M. Reducing ohmic losses in metamaterials by geometric tailoring. *Phys. Rev. B* **80**, 125129 (2009).
2. Guney, D. O., Koschny, Th. & Soukoulis, C. M. Intra-connected three-dimensionally isotropic bulk negative index photonic metamaterial. *Opt. Express* **18**, 12348-12353 (2010).
